# Retinal pigment epithelium drives macrophage migration during *Toxoplasma gondii* infection *in vitro*

**DOI:** 10.1590/0074-02760250141

**Published:** 2026-04-10

**Authors:** Alex Martins Nasaré, Roberto Carlos Tedesco, Paula Andrea Faria Waziry, Lorena de Paula Pantaleon, Esther Lopes Ricci, Luís Antônio Baffile Leoni, André Rinaldi Fukushima, Andres Jimenez Galisteo

**Affiliations:** 1 Universidade de São Paulo, Faculdade de Medicina, Hospital das Clínicas, Laboratório de Investigação Médica em Protozoologia, Bacteriologia e Resistência Antimicrobiana-LIM-49, São Paulo, SP, Brasil; 2 Universidade Federal de São Paulo, São Paulo, SP, Brasil; 3 Florida Atlantic University, Schmidt College of Science, Boca Raton, FL, USA; 4 Universidade Presbiteriana Mackenzie, São Paulo, SP, Brasil; 5 Universidade Santo Amaro, São Paulo, SP, Brasil; 6 Universidade de São Paulo, Faculdade de Ciências da Saúde, São Paulo, SP, Brasil

**Keywords:** ocular toxoplasmosis, retinal pigment epithelium, macrophages, cell movement, cytokines, Toxoplasma gondii

## Abstract

**BACKGROUND:**

Ocular toxoplasmosis is a leading cause of infectious posterior uveitis worldwide. The retinal pigment epithelium (RPE), a key barrier and immunomodulatory layer in the eye, is directly targeted by *Toxoplasma gondii* during infection. However, its role in orchestrating the local immune response remains unclear.

**OBJECTIVES:**

To investigate whether RPE cells actively drive macrophage migration during *T. gondii* infection *in vitro*, and to identify associated cytokine profiles.

**METHODS:**

Adult retinal pigment epithelial cells (ARPE)-19 and primary RPE cells were exposed to tachyzoites, soluble antigens or conditioned supernatants. Macrophage migration was assessed using Transwell^®^ and under-agar assays. Cytokines were quantified by cytometric bead array.

**FINDINGS:**

Both ARPE-19 and primary RPE exhibited chemotaxis toward parasite antigens (0.12 - 0.5 μg), and enhanced interleukin-6 (IL-6), IL-10 and tumor necrosis factor-α (TNF-α) secretion. Co-culture with RAW 264.7 macrophages further amplified cytokine production. Primary RPE from infected animals occluded 90% of Transwell^®^ pores within 24h. IL-6 and IL-10 levels strongly correlated with migratory activity (r = 0.82 and 0.77, respectively).

**MAIN CONCLUSIONS:**

RPE cells are not passive targets but active participants in the ocular immune response to *T. gondii.* By secreting IL-6 and IL-10, they establish a chemotactic environment that recruits macrophages. These insights identify the RPE-cytokine-macrophage axis as a potential therapeutic target in ocular toxoplasmosis.

The retinal pigment epithelium (RPE) is a monolayer of pigmented, highly specialized cells that absorbs excess light, recycles visual metabolites and sustains the photoreceptor layer by secreting trophic factors that preserve the choriocapillaris and the outer blood-retinal barrier.^1^ ^,^ ^2^ By modulating antigen presentation and producing immunosuppressive mediators, the RPE also contributes decisively to the immune privilege of the eye.^3^ ^,^ ^4^ ^,^ ^5^ Moreover, RPE cells exhibit interferon-induced antimicrobial responses.^4^

*Toxoplasma gondii* is an obligate intracellular protozoan that infects approximately one-third of the global population and is capable of invading the central nervous system and ocular tissues.^6^ In the eye, the parasite most frequently induces posterior uveitis with chorioretinitis and disruption of the chorioretinal interface,^7^ which may progress to necrotizing retinitis and involve the choroid, vitreous or anterior chamber.^8^ ^,^ ^9^ ^,^ ^10^ Disease severity varies according to host immunity, parasite genotype and genetic background, whereas prevalence correlates with sanitation and dietary habits.^11^ Human infection is usually acquired through ingestion of oocyst-contaminated water or food — particularly raw or undercooked meat — or congenitally via transplacental transmission.^6^ In addition, extracellular vesicles released by *T. gondii* can trigger host immune modulation.^12^

Following ingestion, bradyzoites differentiate into tachyzoites that infect circulating monocytes, facilitating hematogenous dissemination to immune-privileged organs such as the brain and eye. Experimental evidence demonstrates that RPE cells enable the transmigration of infected monocytes across the blood-retinal barrier, after which the parasite invades resident ocular cells; subsequent RPE migration towards infected retinal foci in *in vivo* models further disrupts retinal architecture.^10^ Neutrophil-adult retinal pigment epithelial cells (ARPE-19) interactions have also been implicated in ocular toxoplasmosis.^13^ Ultrastructural studies indicate that *T. gondii* manipulates host cell organelles to secure metabolic substrates^14^ and modulates host-cell signaling pathways to evade immune responses,^15^ indicating direct parasite-RPE communication. These findings are consistent with previous reviews describing immune subversion mechanisms employed by *T. gondii*.^16^ Pro-inflammatory cytokines such as tumor necrosis factor-α (TNF-α) and interferon-γ (IFN-γ) further modulate RPE infection dynamics.^1^

Previous studies have demonstrated that ARPE-19 cells recognize and migrate towards *T. gondii*-infected cells *in vivo*,^17^ suggesting an active defensive role for the retinal epithelium. The present study therefore investigates the mechanisms by which the RPE — using both the ARPE-19 cell line and primary retinal pigment epithelial (pRPE) cells — senses *T. gondii* infection and directs macrophage recruitment. The aim is to clarify how these interactions shape the pathogenesis of ocular toxoplasmosis and to identify potential therapeutic targets. Supporting this concept, recent *in vivo* transcriptomic and proteomic analyses have revealed extensive host modulation during infection.^18^ Furthermore, exposure to *T. gondii* upregulates innate immunity and cytokine-related pathways in human RPE cells, particularly interleukin-6 (IL-6)/STAT3-dependent signaling,^15^ ^,^ ^19^ reinforcing the concept of an active immunoregulatory role for these cells.

Based on these observations, we hypothesized that RPE cells (ARPE-19 and pRPE), when exposed to *T. gondii* antigens, actively secrete IL-6 and IL-10, thereby establishing a chemotactic gradient that recruits macrophages and modulates the local inflammatory response.

## MATERIALS AND METHODS

*ARPE-19 cell culture* - The immortalized human retinal pigment epithelial cell line ARPE-19 (ATCC CRL-2302) was kindly provided by the CASO Laboratory, Federal University of São Paulo, Brazil. Cells were maintained in Dulbecco’s Modified Eagle Medium/Nutrient Mixture F-12 (DMEM/F-12; Thermo Fisher Scientific) supplemented with 10% (v/v) heat-inactivated fetal bovine serum (FBS; Gibco), 100 U mL⁻¹ penicillin and 100 μg mL⁻¹ streptomycin, at 37ºC in a humidified atmosphere containing 5% CO₂, as previously described.^2^ Subconfluent monolayers were detached using 0.05% trypsin-EDTA and reseeded as required, including on Transwell® inserts for migration assays.

*Experimental infection of ARPE-19 cells* - ARPE-19 cultures were infected with the type II *T. gondii* ME-49 strain at a concentration of 6 × 10⁵ tachyzoites mL⁻¹, corresponding to a multiplicity of infection (MOI) of 3:1. Cultures were incubated under the same conditions as uninfected controls. After 24 h, the culture medium was replaced with fresh complete DMEM/F-12. Infected cultures were passaged at least four times and are hereafter referred to as sensitized ARPE-19 (ARPE-19-S). This experimental design was used to model immunologically primed epithelial cells and to approximate the immune activation observed in pRPE cells obtained from infected animals.

*Preparation of parasite antigen and conditioned supernatant* - The RH strain of *T. gondii* was used for antigen preparation owing to its high tachyzoite yield, whereas the cyst-forming ME-49 strain was employed for infection assays to better reproduce features of chronic infection. At 24 h post-infection, cell-free supernatants were collected, clarified by centrifugation at 3,000 × g for 10 min at 4ºC, filtered through 0.22 μm membranes and stored at -20ºC until use.

*Experimental animals* - Twenty male C57BL/6J mice (eight weeks old; 20 ± 2 g) were obtained from the animal facility of the University of São Paulo Medical School. All experimental procedures were approved by the local ethics committee (CEUA-IMT/USP, protocol 000349A) and were conducted in accordance with the ARRIVE 2.0 guidelines.^20^ Animals were acclimatized for 45 days and allocated into three experimental groups: (i) control (no intervention); (ii) immunized, receiving three intraperitoneal doses of 1 × 10⁴ γ-irradiated ME-49 tachyzoites at 15-day intervals; and (iii) infected, receiving a single intraperitoneal dose of 1 × 10³ viable ME-49 tachyzoites 30 days prior to sample collection.

*pRPE cells* - Primary mouse retinal pigment epithelial cells were isolated according to previously described protocols.^21^ ^,^ ^22^ Briefly, neuroretinas were removed and eyecups were incubated in 0.2% collagenase D (Promega) diluted in DMEM for 45 min at 37ºC. Dissociated cells were cultured in complete DMEM/F-12 medium, seeded at a density of 2.5 × 10⁵ cells per well and used up to the second passage. Culture medium was renewed every 48 h.

*ARPE-19 and RAW 264.7 co-culture* - Murine macrophages (RAW 264.7; ATCC TIB-71) were counted using a Neubauer chamber and co-cultured with ARPE-19 cells at a ratio of 1 × 10⁵ to 5 × 10⁵ cells (RAW:ARPE). After 24 h, five experimental conditions were established: RAW macrophages alone, ARPE-19 with RAW macrophages, sensitized ARPE-19 with RAW macrophages, ARPE-19 alone and sensitized ARPE-19 alone. Co-cultures were infected with the *T. gondii* RH strain as described above. Supernatants were collected 24 h post-infection and stored at -80ºC until analysis.

*Migration assays / Transwell® assay* - ARPE-19 or pRPE cells (2.5 × 10⁵ cells per insert) were seeded onto polyester Transwell® inserts with 8.0 μm pores (Corning) and cultured in complete medium for 24 h. After reaching confluence, the medium was replaced with serum-free DMEM/F-12 and the appropriate stimulus — parasite antigen, conditioned supernatant or live tachyzoites — was added to the lower chamber. After 24 h, supernatants were collected and stored at -80ºC. Cells remaining on the upper surface of the membrane were removed with cotton swabs, and membranes were fixed in 2.5% glutaraldehyde in phosphate-buffered saline (PBS) for light microscopy analysis. Cell viability was assessed before and after each assay using Trypan Blue exclusion, with viability consistently exceeding 95%. All experiments were performed using ten independent biological replicates with technical triplicates.

*Under-agar assay* - Under-agar migration assays were performed as previously described.^23^ A 1% (w/w) agar solution in PBS was autoclaved, mixed 1:1 with pre-warmed complete DMEM/F-12 and poured into six-well plates (8 mL per well). After solidification, three parallel wells (7.0 mm diameter) were created in the agar. Migration towards 10% FBS was used as a positive chemotactic control, while serum-free DMEM/F-12 served as a negative control. The central well was loaded with ARPE-19 or sensitized ARPE-19 cells (3.8 × 10² cells mm⁻²), while lateral wells contained either control media, serial dilutions of RH antigen (0.12 - 2.0 μg), conditioned supernatant or viable tachyzoites. After 24 h, supernatants were collected for cytokine analysis. Cells were then fixed in 4% paraformaldehyde, stained with DAPI and imaged by fluorescence microscopy. Migration distances were quantified using ImageJ (FIJI version).

*Microscopy / light microscopy* - Fixed Transwell® membranes were dehydrated in graded ethanol solutions, infiltrated overnight with a mixture of Technovit 7100 (glycol methacrylate) and ethanol (1:1), embedded in resin, sectioned at 1 μm thickness and stained with 0.25% toluidine blue in 1% sodium borate. Sections were mounted using Erv-Mont™ mounting medium and examined under a Nikon Eclipse light microscope.

*Transmission electron microscopy* - Confluent pRPE monolayers grown on 0.8 μm-pore inserts were serum-starved for 24 h and exposed to 1.2 × 10⁵ ME-49 tachyzoites added to the upper compartment without direct cell contact. Pre- and post-exposure supernatants were collected and stored at -80ºC. Cells were fixed in 2.5% glutaraldehyde, post-fixed in 1% osmium tetroxide, dehydrated, embedded in epoxy resin, ultrathin-sectioned and examined using a JEOL JEM-1010 transmission electron microscope.

Environmental factors influencing cytokine signaling in retinal pigment epithelial cells were considered during experimental design.^24^

*Cytokine quantification* - Supernatants were diluted 1:1 in BD FACSFlow™ sheath fluid and analyzed using a BD LSRFortessa flow cytometer with the BD Cytometric Bead Array Mouse Cytokine Kit, following the manufacturer’s instructions. Data acquisition was performed using FACSDiva software version 8.0 and analyzed with FlowJo version 10.8.

*Statistical analysis* - Statistical analyses were performed using GraphPad Prism version 9.0 (GraphPad Software, San Diego, CA, USA). Group means were compared using one-way analysis of variance (ANOVA) followed by Tukey’s post-hoc test. Values of p < 0.05 were considered statistically significant. Effect sizes were calculated using Cohen’s d and interpreted as low (d < 0.5), medium (0.5 ≤ d < 0.8) or high (d ≥ 0.8). All raw data, processed images and analysis scripts are available from the corresponding author upon reasonable request.

## RESULTS

**Fig. 1:**
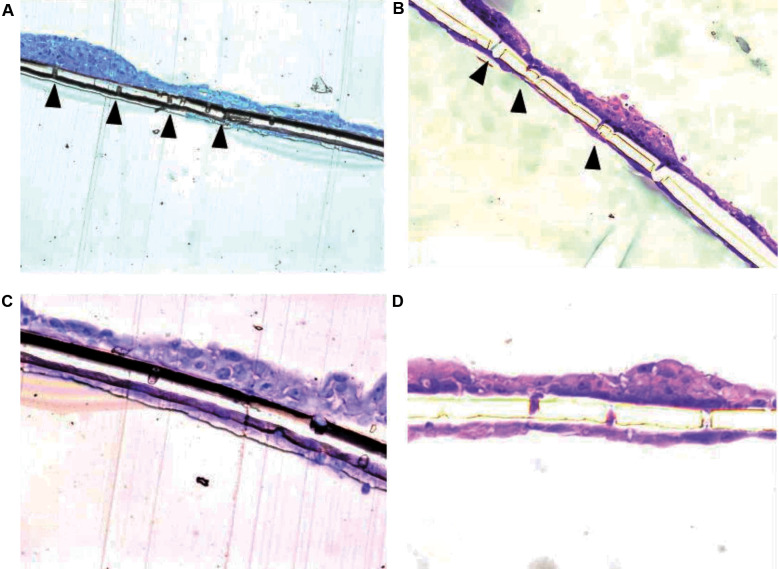
transmembrane migration of adult retinal pigment epithelial (ARPE-19) cells through Transwell® insert pores to the lower surface of the membrane. (A, B) ARPE-19 cells showing gaps (arrows) corresponding to pores on the lower membrane surface without cell coverage. (C, D) Sensitised ARPE-19 cells (ARPE-19-S) forming a continuous monolayer completely covering the pores.

**Fig. 2:**
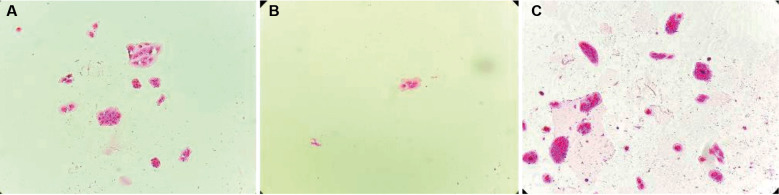
Transwell® migration assay showing adult retinal pigment epithelial (ARPE-19) cell migration through membrane pores and adhesion to the lower surface of the well. (A) Control condition. (B) Exposure to *Toxoplasma gondii* RH strain (2.5 × 10⁻³ μg/μL). (C) Exposure to soluble *T. gondii* antigen. Both stimuli increased the migratory response.

*ARPE-19 migration in Transwell® assays* - Both non-infected ARPE-19 and sensitized ARPE-19 (ARPE-19-S) monolayers were able to traverse the 8.0 μm-pore Transwell® membranes. On the lower surface of the membranes, ARPE-19-S cells formed a continuous layer completely covering the pores, whereas ARPE-19 cells displayed focal gaps corresponding to pores devoid of cells (Fig. 1A-D). Quantitative analysis of pore occupancy confirmed a significantly higher proportion of fully occluded pores in ARPE-19-S cultures compared with ARPE-19 cultures (p < 0.05). Migrated ARPE-19 cells were also observed adhering to the lower surface of the insert membrane and, in some cases, detaching and settling at the bottom of the well (Fig. 2A-C).

**Fig. 3:**
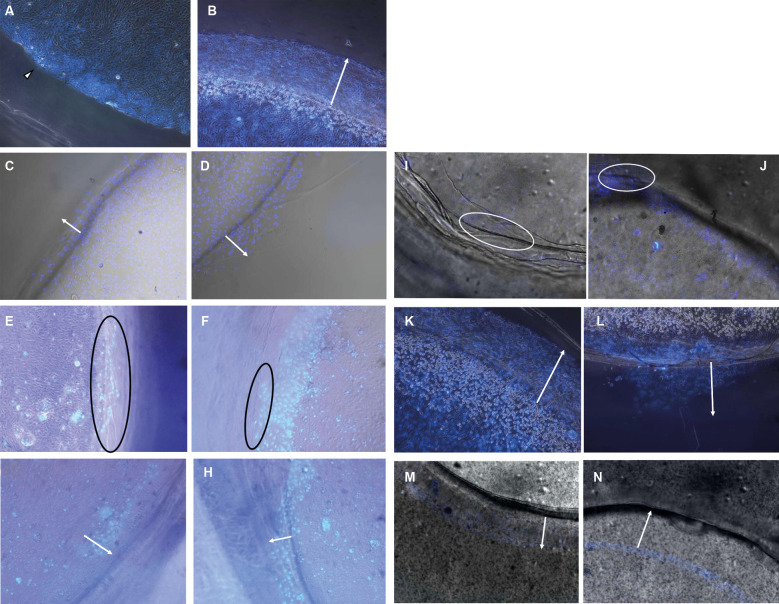
chemotaxis of adult retinal pigment epithelial (ARPE-19) and sensitised ARPE-19 cells in the under-agar assay after 24 h. Representative images under different experimental conditions. Arrows indicate the migration front.

**Fig. 4:**
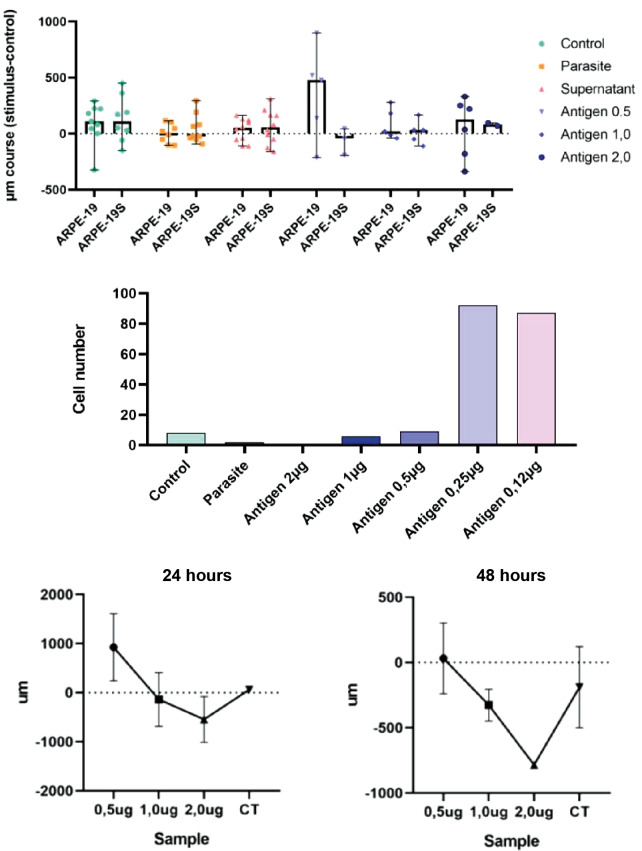
quantification of under-agar migration. (A) Median migration distance after 24 h. (B) Number of cells crossing the agar interface. (C) Comparison of migration at 24 h and 48 h. Values are mean ± standard deviation (SD) (n = 5).

**Fig. 5:**
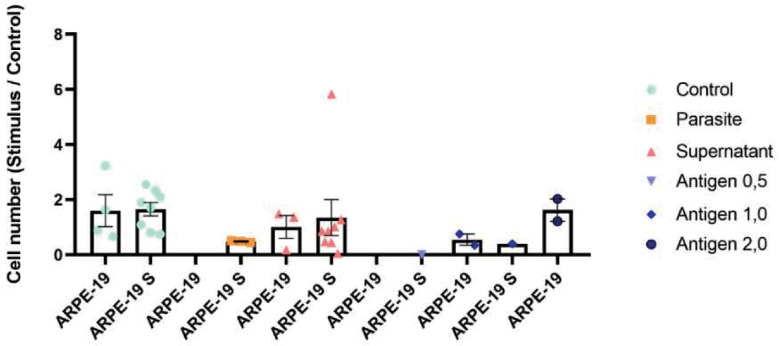
quantification of adult retinal pigment epithelial (ARPE-19) cell migration in the Transwell® assay after exposure to different stimuli. Bars represent the number of cells in the lower chamber following exposure to live tachyzoites, conditioned supernatant or soluble antigen (0.12-2.0 μg). Values are mean ± standard deviation (SD) (n = 3). *p < 0.05 versus control [analysis of variance (ANOVA)/Tukey].

*Chemotaxis measured by the under-agar assay* - ARPE-19 cell migration in the under-agar assay exhibited a clear dose-dependent pattern. Migration distances were greatest at lower concentrations of soluble *T. gondii* antigen (0.12-0.50 μg), with median distances exceeding 500 μm after 24 h. At higher antigen concentrations (1.0-2.0 μg), migration was markedly reduced and approached values observed under negative control conditions (Figs 3A-L, 4A). Neither conditioned supernatants nor live tachyzoites induced measurable directional migration. The total number of cells crossing the agar interface did not differ significantly among experimental groups (Figs 4B, 5). Prolongation of the incubation period to 72 h did not alter the observed migration profile (Fig. 4C).

*Cytokine secretion by ARPE-19 cultures* - Supernatants collected from Transwell® migration assays contained detectable levels of IL-6, IL-10 and TNF-α. Peak concentrations of these cytokines coincided with the antigen concentrations (0.12-0.50 μg) that induced maximal migratory responses. Strong positive correlations were observed between migration distance and cytokine levels for IL-6 (r = 0.82, p < 0.01), IL-10 (r = 0.77, p < 0.05) and TNF-α (r = 0.74, p < 0.05; Pearson correlation). IL-2 was detected predominantly under non-migratory conditions and was absent in sensitized ARPE-19 cultures exposed to live tachyzoites, suggesting that IL-2 release was not associated with chemotactic activity (Table I).

**TABLE I:** Cytokines secreted by adult retinal pigment epithelial (ARPE-19) and sensitised ARPE-19 cells (ARPE-19-S) after stimulation. Relative expression (z-scores) of interleukin-6 (IL-6), IL-10, tumour necrosis factor-alpha (TNF-α) and IL-2 following exposure to live *Toxoplasma gondii* tachyzoites, conditioned supernatant or soluble antigen (0.12-2.0 μg). Green indicates values approaching 1, and red indicates values approaching -1

		Parasite	Supernatant	0.5 µg	1 µg	2 µg	0.25 µg	0.125 µg
IL-6	ARPE-19	0.8164	0.8164	0.8164	0.8164	0.6237	0.8164	0.8164
	ARPE-19 S	0.8165	0.8165	0.8165	0.8165	0.8165	-1.1466	0.8165
IL-10	ARPE-19	0.8793	0.8793	0.8793	0.8793	0.8793	0.7049	0.6762
	ARPE-19 S	0	0	0	0	0	0	-0.8164
TNF-α	ARPE-19	0.8164	0.8164	0.8164	0.3495	0.8164	0.8164	0.8164
	ARPE-19 S	0	0	0	0	0	-0.8164	0
IL-2	ARPE-19	0.8391	0.8672	0.8774	1.2624	0.8112	0.5896	0.8515
	ARPE-19 S	1.2838	1.2838	1.2838	1.035	0.9979	0.6928	0.9137

	1	0.8	0.6	0.4	0.2	0	-0.2	-1

*Cytokine profile of ARPE-19-RAW 264.7 co-cultures* - Exposure of ARPE-19 cells to *T. gondii* tachyzoites resulted in approximately twofold increases in IL-6 and IL-10 secretion. Co-culture with RAW 264.7 macrophages markedly amplified cytokine production, with IL-6 concentrations reaching up to 362 pg mL⁻¹ and IL-10 levels up to 343 pg mL⁻¹. Infected co-cultures exhibited even higher cytokine levels, producing up to 842 pg mL⁻¹ IL-6 and 471 pg mL⁻¹ IL-10, along with substantial TNF-α secretion (742 pg mL⁻¹). Effect size analysis using Cohen’s d classified the increases in IL-6, IL-10 and TNF-α as high across parasite-stimulated co-culture conditions (Table II).

**TABLE II:** Cytokines production by adult retinal pigment epithelial (ARPE-19) and sensitised ARPE-19 cells (ARPE-19-S) stimulated with *Toxoplasma gondii* and/or RAW 264.7 macrophages. Interleukin-6 (IL-6), IL-4, IL-10, interferon-gamma (IFN-γ), and tumour necrosis factor-alpha (TNF-α) levels were quantified using cytometric bead array (CBA). The impact of exposure was calculated using Cohen’s d and classified as null, low, medium or high. Values represent the meaning of three independent experiments

		Control		Parasite		Size effect	D de Cohen
IL-6	RAW	0	0	0	0	0	Null
	RAW + ARPE - 19	0	0.41	362.13	0.7	1	High
	RAW + ARPE - 19 S	5.46	3.31	841.97	1983.91	2	High
	ARPE - 19	0	1.88	0.6	0	0.6	Medium
	ARPE - 19 S	0	0.11	2.28	0	0.9	High
IL-4	RAW	0	0	0	0	0	Null
	RAW + ARPE - 19	0.53	1.4	0.95	1.29	0.33	Low
	RAW + ARPE - 19 S	0.35	0	0	1.15	0.66	Medium
	ARPE - 19	0.3	3.42	0	0	1.19	High
	ARPE - 19 S	0	0.39	0.76	0	0.4	Low
IL-10	RAW	0	0	0	0	0	Null
	RAW + ARPE - 19	1.61	0	343.35	247,53	6,14	High
	RAW + ARPE - 19 S	12,45	3,81	471,43	332,94	5,67	High
	ARPE - 19	0	17,74	1,61	0	0.9	High
	ARPE - 19 S	0	0	4.05	0	1	High
IL-2	RAW	0	0	0	0	0	Null
	RAW + ARPE - 19	1.02	0.74	1.33	0.87	0.8	High
	RAW + ARPE - 19 S	0	1.08	1.38	0.72	0.8	High
	ARPE - 19	1.08	2.38	1.29	0.51	1.09	High
	ARPE - 19 S	0	1.73	0	0	1	High
TNF-α	RAW	0	0	0	0	0	Null
	RAW + ARPE - 19	270.34	350.5	400.2	27.78	0.5	Medium
	RAW + ARPE - 19 S	2423.33	2290.84	742.25	1152.09	6.5	High
	ARPE - 19	0	11.96	0	0	1	High
	ARPE - 19 S	5.79	0	1.88	0	0.64	Medium

0	1	2	3	4	5	6	7	8	9	10

All numerical data are presented as mean ± standard deviation (SD) (n = 5). Statistical comparisons were performed by one-way analysis of variance (ANOVA) followed by Tukey’s post-hoc test; *p < 0.05.

**Fig. 6:**
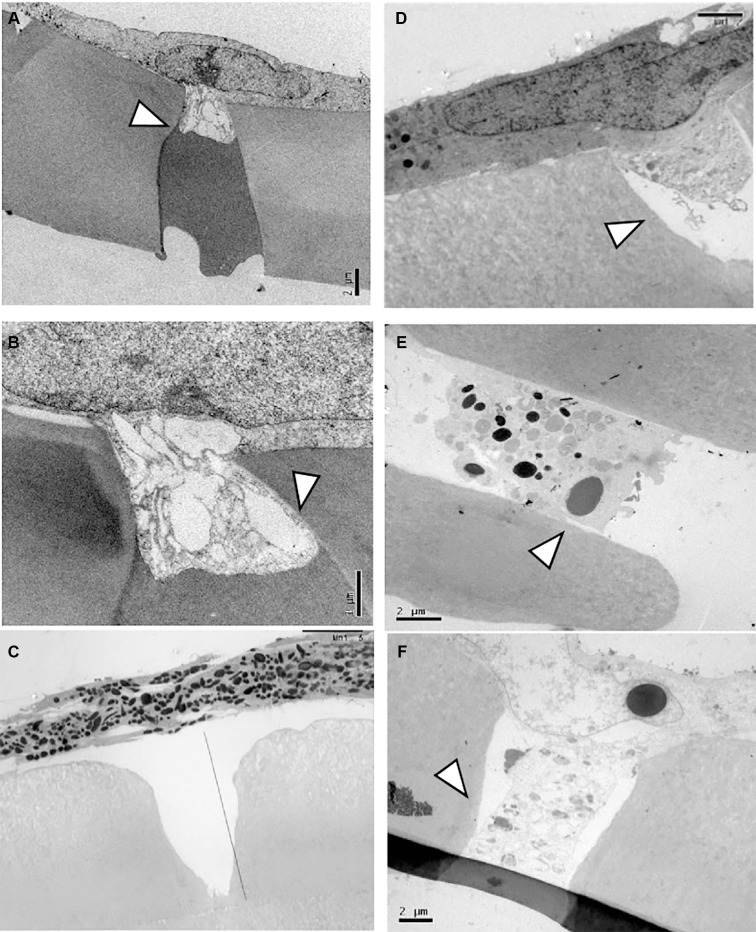
migration of primary retinal pigment epithelial (pRPE) cells analysed by transmission electron microscopy. (A, B) Cells from control animals. (C, D) Cells from immunised animals showing membrane protrusions traversing the pores (arrows). (E, F) Cells from infected animals showing complete pore occlusion and disrupted membranes.

*Migration of pRPE cells* - pRPE cells isolated from control mice exhibited limited migratory activity and preserved plasma membrane integrity, with partial pore occupancy observed after 24 h (Fig. 6A). Cells derived from immunized animals occupied a larger proportion of Transwell® membrane pores and displayed numerous cytoplasmic vesicles and membrane protrusions (Fig. 6B-C). In contrast, pRPE cells from infected animals showed extensive membrane disruption and virtually complete pore occlusion, indicating intense migratory activity (Fig. 6D-F).

**Fig. 7:**
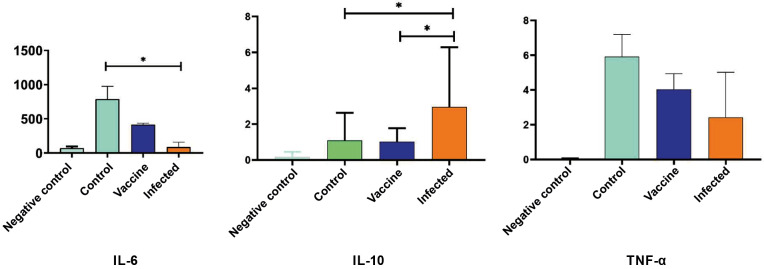
cytokine secretion by primary retinal pigment epithelial (pRPE) cells after exposure to *Toxoplasma gondii* ME-49 strain. (A) interleukin (IL)-6. (B) IL-10. (C) tumour necrosis factor-alpha (TNF-α). Groups: basal, control, immunised and infected. Values are expressed as pg mL⁻¹ [mean ± standard deviation (SD), n = 3]. *p < 0.05 [analysis of variance (ANOVA)/Tukey].

*Cytokine secretion by pRPE cells* - Basal pRPE cultures secreted high levels of IL-6 (788 pg mL⁻¹), low levels of TNF-α (5.9 pg mL⁻¹) and no detectable IL-10. pRPE cells from immunized animals produced lower IL-6 concentrations (414 pg mL⁻¹) while maintaining similar TNF-α levels. Infected pRPE cultures secreted markedly reduced IL-6 levels (153 pg mL⁻¹) but exhibited the highest IL-10 concentrations (6.6 pg mL⁻¹). TNF-α concentrations remained relatively constant across all experimental groups (approximately 6 pg mL⁻¹) (Fig. 7A-C).

## DISCUSSION

Ocular toxoplasmosis remains one of the leading infectious causes of posterior uveitis and vision loss worldwide, yet the cellular events that initiate retinal damage remain poorly defined. The present study demonstrates that human and murine RPE actively detects and responds to *T. gondii*, orchestrating a chemotactic axis dominated by IL-6 and IL-10 *in vitro*. Three key findings emerge from our results.

*RPE is intrinsically migratory and dose-responsive* - ARPE-19 and pRPE cells migrated through Transwell® membranes and under-agar gradients, but only when exposed to soluble *T. gondii* antigen concentrations ≤ 0.5 μg. Higher doses abolished chemotaxis, indicating a bell-shaped dose-response curve similar to that described for professional phagocytes.^13^ These data extend previous *in vivo* findings showing that RPE cells migrate toward intraretinal parasites^17^ ^,^ ^25^ and confirm that this response can be reliably reproduced *in vitro*. Comparable IL-6/IL-10-driven communication between epithelial and macrophage compartments has been observed in intestinal and pulmonary tissues,^26^ suggesting that RPE cells share conserved immunomodulatory mechanisms with other mucosal barriers. Similar transcriptional changes in human RPE during *T. gondii* infection have also been documented.^18^

*IL-6, IL-10 and TNF-α correlate with migration intensity* - Soluble antigen concentrations that maximized chemotaxis also induced the greatest secretion of IL-6, IL-10 and TNF-α, whereas IL-2 was associated with non-migratory conditions.^22^ Co-culture with RAW 264.7 macrophages amplified IL-6, IL-10 and TNF-α release, reinforcing the dynamic communication between retinal epithelial cells and macrophages.^27^ ^,^ ^28^ ^,^ ^29^ IL-6 may act as an autocrine primer that upregulates IL-1 expression, providing negative feedback to limit tissue damage.^2^ ^,^ ^16^ The moderate TNF-α concentrations detected are consistent with a local, protective response that restrains parasite replication without compromising RPE integrity.^19^ ^,^ ^21^

*Prior exposure to T. gondii primes pRPE motility* - pRPE cells from immunized or chronically infected mice occluded nearly every Transwell® pore within 24 h, whereas control cells required at least 48 h. This priming was accompanied by elevated IL-10 levels in infected animals and sustained IL-6 production in control cells, as previously described in experimental models of *T. gondii* infection,^21^ suggesting that systemic antigen exposure conditions the ocular microenvironment,^5^ thereby accelerating pRPE chemotaxis during subsequent challenges.^15^ These cytokine profiles closely resemble those reported in recurrent toxoplasmic retinochoroiditis.^30^

*Proposed model* - Upon retinal exposure to low concentrations of *T. gondii* antigen, RPE cells secrete IL-6 and TNF-α, initiating a controlled pro-inflammatory milieu that attracts macrophages and promotes their own migration toward the infectious focus. As IL-10 levels rise, this cytokine attenuates IL-6 signaling, preserving retinal architecture yet inadvertently favoring parasite persistence, a balance that may underlie recurrent clinical relapses.^14^ Conversely, excessive antigen exposure or an overwhelming tachyzoite load disrupts this regulatory axis, suppressing chemotaxis and potentially permitting uncontrolled parasite dissemination.

*Study limitations and future directions* - The present study relied exclusively on *in vitro* assays, which may not fully recapitulate the *in vivo* retinal microenvironment. Validation using retinal organoids or animal models will therefore be crucial to confirm the physiological relevance of the IL-6/IL-10 axis. The ARPE-19 cell line, although widely used,^7^ ^,^ ^26^ does not fully reproduce the polarity or phagocytic capacity of primary RPE cells.^31^ Only early-passage primary cells were analyzed to minimize culture artefacts; however, limited yields constrained the performance of more detailed mechanistic assays. Future investigations should employ organoid or explant models to verify cytokine gradients *in situ*, as previously explored in experimental infection models,^27^ dissect downstream signaling pathways — particularly STAT3- and STAT6-dependent signaling — and assess whether pharmacological modulation of this axis mitigates experimental ocular toxoplasmosis.

*Clinical implications* - These findings open new avenues for the therapeutic modulation of cytokine balance in ocular toxoplasmosis, offering opportunities for integrated immunomodulatory and antiparasitic strategies. Targeting the IL-6/IL-10 axis may provide a dual advantage by enhancing parasite clearance while minimizing collateral retinal injury. Agents that transiently enhance IL-6 signaling or antagonize IL-10 at early stages might strengthen host defenses, whereas sustained IL-6 inhibition could prevent chronic inflammation once parasite burden is controlled. Such stage-specific interventions warrant evaluation in preclinical models.

*Concluding remarks* - This study demonstrates, for the first time, that RPE cells exhibit a dose-dependent chemotactic response to *T. gondii* antigens, governed by the coordinated action of IL-6 and IL-10. These findings establish a mechanistic link between epithelial activation and macrophage recruitment in ocular toxoplasmosis, identifying a potential therapeutic target for the modulation of retinal inflammation. This migratory behavior is accompanied by a cytokine profile dominated by IL-6 and TNF-α, subsequently attenuated by IL-10. Collectively, the results support a model in which ARPE-19 cells and pRPE cells detect the parasite, initiate a controlled pro-inflammatory response to recruit immune cells, and later limit tissue damage through IL-10-mediated feedback. Targeted modulation of the IL-6/TNF-α/IL-10 axis therefore emerges as a promising strategy to mitigate retinal injury while preserving host defenses in ocular toxoplasmosis.

The authors declare no competing interests.

## Data Availability

All data generated and analysed during this study are included in the manuscript or are available from the corresponding author upon reasonable request.
